# Impact of pectoralis muscle loss on cardiac outcome and survival in Cancer patients who received anthracycline based chemotherapy: retrospective study

**DOI:** 10.1186/s12885-022-09882-w

**Published:** 2022-07-13

**Authors:** Wael Toama, Jason Wiederin, Ryan Shanley, Patricia Jewett, Christina Gu, Chetan Shenoy, Prabhjot S. Nijjar, Anne H. Blaes

**Affiliations:** 1 Division of Hematology, Oncology and Transplantation, University of Minnesota, 420 Delaware Street SE, MMC 480, 14-142E Phillips-Wangensteen Bldg, Minneapolis, MN 55455 USA; 2 Department of Medicine, Hennepin Healthcare, Minneapolis, MN USA; 3 University of Minnesota Masonic Cancer Center, Biostatistics Core, Minneapolis, MN USA; 4 University of Minnesota Medical School, Minneapolis, MN USA; 5 Division of Cardiology, University of Minnesota, Minneapolis, MN USA

**Keywords:** Sarcopenia, Cardiac, Breast cancer, Lymphoma, Sarcoma

## Abstract

**Introduction:**

The impact of pectoralis muscle mass index (PMI) on cardiac events is not well studied in cancer patients, especially in those who have received chemotherapy with high potential cardiac toxicity such as anthracyclines.

**Methods:**

Individuals aged ≥18 years with a diagnosis of breast cancer, sarcoma, or lymphoma who received anthracycline-based chemotherapy at the University of Minnesota MHealth Fairview between 2009 and 2014. Eligible patients had to have two CT scans: a baseline CT scan within 6 months prior to chemotherapy and a follow-up CT scan within 2 years after treatment. The PMI was calculated as the right pectoralis muscle area indexed to height squared. Multivariable linear regression was used to analyze factors associated with PMI at follow-up, overall mortality, and major cardiac events (MACE).

**Results:**

A total of 474 patients (breast cancer 192; lymphoma 184; sarcoma 98) participated with a median age of 61 years at the time of baseline CT scan; 161 (34%) were male. Almost all patients received anthracyclines except 12% who received trastuzumab only. The median baseline PMI was 5.8 cm^2^/m^2^ (4.9, 7.7) which decreased 10.5% after chemotherapy, to 5.2 cm^2^/m^2^ (4.4, 6.4). Baseline PMI was not significantly associated with OS, but we detected lower risks of MACE with larger PMI at baseline. Greater baseline PMI was associated with greater follow-up PMI, but also with greater relative PMI loss. Female gender, older age, and history of smoking were also associated with greater PMI losses.

**Conclusion:**

Greater pre-treatment pectoralis muscle index in patients treated with anthracyclines have a lower risk of MACE. Early identification of sarcopenia using PMI could trigger proactive engagement for intervention and risk-stratified therapies.

**Supplementary Information:**

The online version contains supplementary material available at 10.1186/s12885-022-09882-w.

## Introduction

Sarcopenia was first described in 1989 to characterize lean muscle losses that accompany aging [1]. Over the last decades, sarcopenia has become a viable predictor of adverse outcomes in multiple types of cancer; these outcomes are related to the diagnosis itself, cancer treatment and its complications [2–4]. There is no universal consensus on the definition of sarcopenia or the best methodology or technique to assess it. The most widely accepted definition has broadened to not only involve low muscle mass, but also reduced strength and/or physical performance [5]. The increased routine use of CT scan in many cancer types for staging, monitoring and surveillance makes this technique attractive to appraise muscle mass and body composition [6].

Pectoralis muscle area has been used as a surrogate for whole muscle mass. Other muscles are also used such as total abdominal muscle area at the third lumbar vertebra on the axial view of a CT scan [7–9].

Pectoralis muscle area, however, has been demonstrated to be both an independent marker of muscularity and a predictive marker of overall survival (OS) in patients with non-small cell lung cancer [10]. Higher baseline pectoralis muscle index (PMI) and baseline pectoralis muscle density have been shown to be associated with better OS in patients with sarcoma [11].

Sarcopenia has been evaluated as a prognostic marker in the field of cardiology. Unilateral pectoralis muscle mass indexed to body surface area calculated preoperatively and indexed to height squared (pectoralis muscle index, PMI; cm^2^/m^2^) is a powerful predictor of mortality in advanced heart failure patients undergoing left ventricular assist device (LVAD) implantation [12].

The impact of sarcopenia on cardiac events is not well studied in cancer patients who have received chemotherapy with high potential cardiotoxicity such as anthracyclines [13]. We investigated the association of pre-chemotherapy pectoralis muscle mass index (PMI) with major cardiac events and overall survival in individuals with cancer receiving anthracyclines, hypothesizing that greater pre-chemotherapy PMI would be associated with improved survival and cardiac outcomes. Additionally, we explored potentially predisposing factors for experiencing PMI loss after chemotherapy.

## Methods

### Study population and data collection

We identified patients aged ≥18 years with a diagnosis of breast cancer, sarcoma, or lymphoma who received anthracyclines and/or trastuzumab-based chemotherapy at the University of Minnesota MHealth Fairview between 2009 and 2014. Eligible patients had to have two CT scans, with a baseline CT scan within 6 months prior to chemotherapy initiation and the second (follow-up) CT scan within 2 years after chemotherapy initiation. All patients with sarcoma or lymphoma had received anthracycline; individuals with breast cancer had received anthracyclines, trastuzumab, or both. Individuals with a cardiac event prior to chemotherapy were excluded. Chart abstraction was performed to confirm specific diagnoses, staging, treatment regimen, duration of therapy, and date of CT scans before and after chemotherapy. The institutional review board of University of Minnesota approved this retrospective study.

### Measurement of pectoralis muscle index (PMI)

We measured the right pectoralis muscle area [cm^2^] at baseline and follow-up CT scans using CORESLICER, a validated web-based application for accurate assessment of muscular body composition [14]. Two readers (JW, CG) reviewed a single axial slice of chest CT above the aortic arch. Once the appropriate axial slice was found, readers used the software’s shading function to estimatearea (cm^2^) of the right pectoralis major and minor muscles. These raw pectoralis measurements were then indexed to the height square to calculate PMI (PMI: pectoralis muscle area [cm^2^]/body height^2^ [m^2^])).

### Primary and secondary outcomes

In our time-to-event analyses, the primary outcomes were 1) death from any cause, and 2) any fatal or non-fatal major cardiac events (MACE). MACE included: Hospitalization or emergency visits for heart failure (HF), outpatient diagnosis of HF, non-fatal MI and cardiovascular death. Our secondary outcome was to explore potential risk factors for declining PMI following chemotherapy such as pre-treatment PMI, sex, smoking history, cancer type, stage, and treatment with anthracycline with or without trastuzumab.

### Statistical analysis

Survival time was calculated as the time between start of chemotherapy and the event of interest (death/cardiac event or censoring at the end of the study with cutoff at September, 12018). In the time-to- event analyses, we included patients who received treatment with curative intent. Therefore, patients with stage IV breast cancer or stage IV sarcoma were excluded from these analyses to avoid bias due to increased mortality in these groups. We used multivariate Cox regression to evaluate factors associated with survival, with baseline PMI (calculated at baseline CT scan) as the primary exposure of interest. We adjusted for age at diagnosis, sex, smoking history, stage (I or II vs. III or IV vs. unknown), and cancer type (breast vs. lymphoma vs. sarcoma). Continuous variables were modeled using 3-knot restricted cubic splines to capture potential non-linear effects. Wald tests were used for null hypothesis testing across the entire range of each variable. For continuous variables, hazard ratios are reported using the 75th and 25th percentile of the distribution as reference values. HRs < 1 indicate protective associations, whereas HRs > 1 indicate greater risk of experiencing the respective events.

For the cardiac event outcome, similar methods were used, except that death without a cardiac event was defined as a competing risk (not censored). Due to the limited number of cardiac events, only baseline PMI, age, and sex were included as covariates. Finally, we added an exploratory unadjusted competing risk analysis, restricting to MACE after the follow-up CT scan in order to avoid potential reverse causality (potential PMI changes after cardiac events); with PMI at baseline and percent change in PMI between the CT scans as exposures in order to explore whether relative change or baseline PMI were more strongly associated with MACE.

We used multiple linear regression to evaluate factors associated with PMI at follow-up CT scan. We included the same covariates as in the survival model, with baseline PMI as main exposure, additionally adjusted for time from start of chemotherapy to follow-up CT scan, and treatment type (anthracyclines, trastuzumab, or both). We used 5-knot restricted cubic splines for continuous variables due to the larger effective sample size. The outcome (PMI at follow-up) was log-transformed to stabilize residual variance. Thus, when transformed back to standard units, regression coefficients represent ratios of expected geometric means. A geometric mean ratio > 1 represents relatively higher expected values. Analysis was performed using R software, version 4.0, including the survival and rms code packages.

## Results

A total of 474 patients were included in our analysis (breast cancer *n* = 192, lymphoma *n* = 184, sarcoma *n* = 98), of which 161 (34%) male and 234 (53%) had stage IV cancer (Table 1). Median age at the time of baseline CT scan was 61 years. Almost all patients received anthracyclines except 12% who had trastuzumab only. Most (91%) of all patients had their follow-up CT scan within a year after starting chemotherapy, and 87% within a year after their baseline CT scan. Median baseline PMI of total cohort was 5.8 cm^2^/m^2^ (4.9, 7.7) which on average decreased 10.5% after chemotherapy, to 5.2 cm^2^/m^2^ (4.4, 6.4). Median baseline PMI was 5.3, 6.5 and 6.7 cm^2^/m^2^ which after chemotherapy decreased to 5.6, 12.3 and 17.9% in Breast cancer, lymphoma and sarcoma respectively.

**Table 1:** Characteristics of the study population, by cancer site, *N* = 474

**Cancer site**	**Breast (*N*** **= 192)**	**Lymphoma (*N*** **= 184)**	**Sarcoma (*N*** **= 98)**	**All (*N*** **= 474)**
**PMI at baseline**				
Median (Q1, Q3)	5.3 (4.7, 6.4)	6.5 (5.1, 8.5)	6.7 (5.3, 8.9)	5.8 (4.9, 7.7)
Range	1.6–11.3	2.3–18.0	2.3–15.8	1.6–18.0
**PMI at follow-up**				
Median (Q1, Q3)	5.0 (4.5, 5.7)	5.7 (4.4, 7.6)	5.5 (4.2, 6.7)	5.2 (4.4, 6.4)
Range	0.8–9.9	1.4–17.3	1.8–13.9	0.8–17.3
**Age at baseline**				
Median (Q1, Q3)	57.8 (51.2, 65.0)	63.1 (55.5, 70.6)	60.9 (52.8, 68.8)	60.6 (53.2, 67.6)
Range	44.5, 92.2	44.6, 86.2	45.0, 85.4	44.5, 92.2
**Years from chemotherapy to follow-up CT**				
0–0.49 years	119 (62%)	135 (73%)	63 (64%)	317 (67%)
0.5–0.99 years	41 (21%)	40 (22%)	31 (32%)	112 (29%)
1.0–2.0 years	32 (17%)	9 (5%)	5 (5%)	45 (13%)
**Sex**				
Female	189 (98%)	79 (43%)	45 (46%)	313 (66%)
Male	3 (2%)	105 (57%)	53 (54%)	161 (34%)
**Smoking**				
Never	107 (56%)	92 (50%)	47 (48%)	246 (52%)
Ever	85 (44%)	92 (50%)	51 (52%)	228 (48%)
**BMI**				
Median (Q1, Q3)	27.3 (23.7, 32.4)	27.2 (24.4, 32.1)	28.9 (25.9, 31.2)	27.4 (24.5, 32.0)
Range	16.4–48.1	17.0–60.1	16.4–43.4	16.4–60.1
**Cancer stage**				
I	25 (13%)	22 (13%)	2 (2%)	49 (11%)
II	50 (26%)	22 (13%)	1 (1%)	73 (17%)
III	37 (19%)	21 (13%)	26 (30%)	84 (19%)
IV	79 (41%)	98 (60%)		234 (53%)
Unknown	1	21	57 (66%)	34
I-IV	191	163	12	440
Total	192	184	86 98	474
**Treatment**				
Anthracyclines only	77 (40%)	184 (100%)	98 (100%)	359 (76%)
Trastuzumab only	58 (30%)	0 (0%)	0 (0%)	58 (12%)
Anthracyclines +Trastuzumab	57 (30%)	0 (0%)	0 (0%)	57 (12%)
**Events types (studied in 325 patients)**	**Breast cancer (112)**	**Lymphoma (184)**	**Sarcoma (29)**	**Total (325)**
Death of all-cause	17 (15%)	62 (34%)	14 (48%)	93 (29%)
MACE	13 (12%)	35 (19%)	0 (0%)	48 (15%)
Pre-treatment LVEF < 55%	5 (4%)	9 (5%)	4 (14%)	18 (6%)

### Time to event analysis

Among 325 patients treated with curative intent, 93 died from all-cause mortality (17, 62, and 14 with breast cancer, lymphoma and sarcoma respectively) with a median follow-up time of 5.5 years. There were 49 MACE, (13, 35, 0) in breast cancer, lymphoma and sarcoma respectively. In the competing risks analysis of death from non-cardiac causes as competing event, the small number of MACE (only 48) limited the number of covariates we could adjust for, therefore only age and sex were included in this regression model.

Baseline PMI was not significantly associated with survival (*p* = 0.65; Table 3) However, we observed a decreasing risk of MACE with larger PMI (HR 0.60 for a PMI of 7.9 [75th percentile] relative to 5.0 [25th percentile]; 95% CI 0.36–1.00, *P* = 0.14). In the exploratory competing risk analysis (MACE after follow-up CT scan, 36 events), greater baseline PMI was associated with lower risk of MACE (per additional unit PMI, HR 0.84, 95% CI 0.73–0.97), but relative PMI change (%) was not associated with risk of MACE independent of baseline PMI. Pre-treatment left ventricle ejection fraction (LVEF) < 55% was associated with higher hazards of MACE, however there were only 18 patients with LVEF < 55% (7/18 had MACE) and it did not seem to confound the relationship of PMI with MACE.

### Predictors of pectoralis muscle mass

**Fig. 1:**
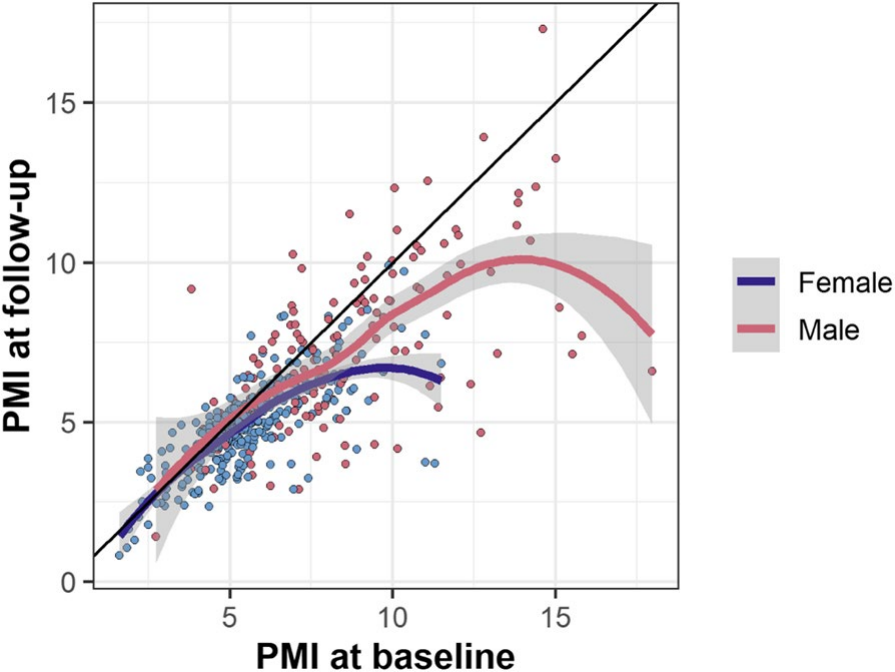
Visualized relationship of PMI at baseline vs. PMI at follow-up, by sex, *N* = 474. Raw values are plotted as dots. Colored lines represent a loess-smoothed trendline for mean follow-up PMI as a function of baseline PMI, unadjusted for other variables, with its 95% confidence interval represented by gray bands

**Fig. 2:**
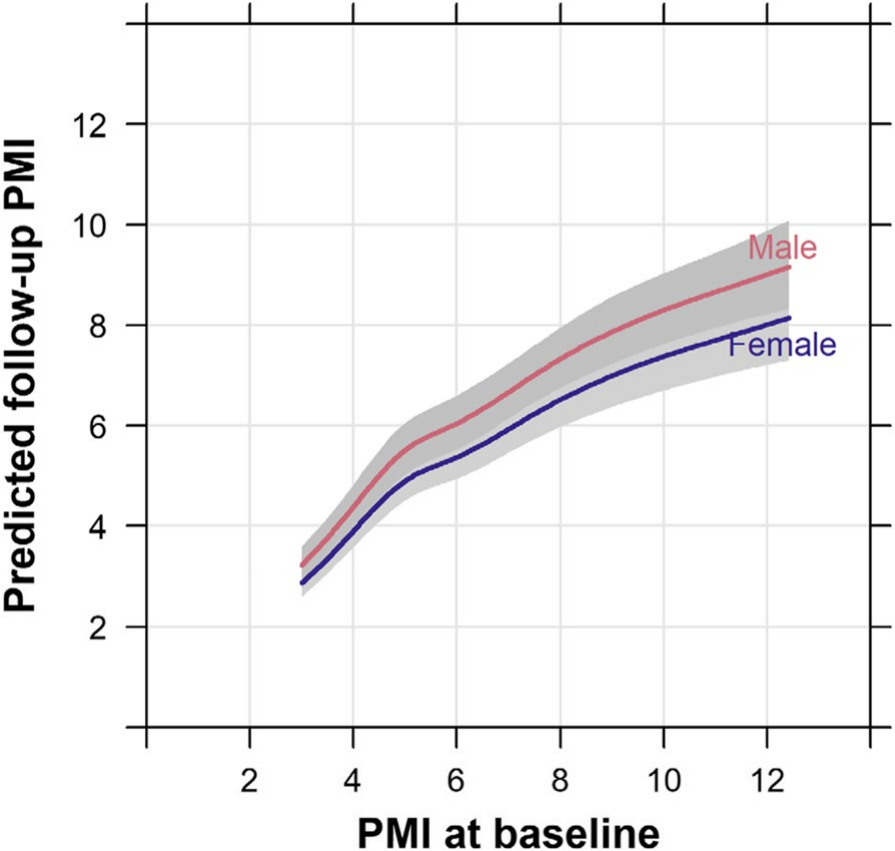
Cubic spline model predicting follow-up PMI as a function of baseline PMI and sex. Adjusted to reference values for age (61 years), stage (1 or 2), smoking history (never) and cancer type (lymphoma). Grey bands represent 95% confidence intervals for mean predicted follow-up PMI

Using PMI (standardized to body height squared) rather than muscle area itself is intended to adjust for systematic differences in muscle mass by sex. However, even after standardization, PMI tended to be higher among males than among females (supplemental Table 1, Fig. 1). As expected, baseline PMI was associated with greater follow-up PMI. We observed a nonlinear relationship, with follow-up PMI on average lower than PMI at baseline (Fig. 2, Table 1). The difference between baseline and predicted follow-up PMI was larger for higher values of baseline PMI, as evidenced by flattening slopes of the lines in Fig. 2. This means that patients with higher PMI at baseline on average lost more muscle after chemotherapy, even though the follow-up PMI remained higher than other patient with lower PMI at baseline. For example, for individuals with a baseline PMI of 8, males had an expected follow-up PMI of 7.4 (8% decrease), and females had an expected follow-up PMI of 6.6 (18% decrease). The geometric mean PMI ratio for males versus females was 1.12 (95% CI: 1.06 to 1.20; Table 2). Since we adjusted for PMI differences at baseline which differed by sex, this describes a sex effect in trajectory, not in the underlying differences in PMI by sex that are due to men having higher PMI in general. Other factors associated with lower follow-up PMI included older age and a history of smoking (Table 2). Notably, we found no evidence for an association between types of chemotherapy (anthracyclines vs. trastuzumab vs. both) with PMI at follow-up.

**Table 2:** Multivariable linear regression of factors associated with PMI at follow-up CT scan, *N* = 474. Reference values for continuous variables are their 75th and 25th percentiles

Characteristic	Geometric mean ratio	95% CI	***P***-value
PMI at baseline [7.7 vs 4.9]	1.32	1.25, 1.40	< 0.01
Age at baseline [67 vs. 53]	0.93	0.87, 0.99	0.01
Years from chemotherapy to follow-up CT [0.6 vs 0.2]	1.03	0.97, 1.09	0.44
Stage			0.91
I or II	1 (Ref.)		
III or IV	1.00	0.95, 1.05	
Unknown	1.04	0.95, 1.14	
Smoking history			< 0.01
Never	1 (Ref.)		
Ever	0.94	0.91, 0.98	
Sex			< 0.01
Female	1 (Ref.)		
Male	1.12	1.06, 1.20	
Cancer Type			0.19
Breast	1 (Ref.)		
Lymphoma	1.00	0.93, 1.07	
Sarcoma	0.95	0.88, 1.02	
Treatment			0.64
Anthracyclines	1 (Ref.)		
Trastuzumab	1.04	0.96, 1.12	
Both	1.01	0.93, 1.09

**Table 3:** Hazard ratios (HR) of all-cause mortality and having a major cardiac event, Survival analysis, *N* = 325 (stage IV and unknown stage of breast cancer and sarcoma excluded); and exploratory analysis of major cardiac events (MACE) after follow-up CT scan, *N* = 306 (patients with MACE prior to follow-up CT scan excluded)

Parameter	Model 1^a^ (All-cause mortality) HR (95% CI)	Model 2^b^ (MACE) HR (95% CI)	Model 3^b**,c**^ (MACE) HR (95% CI)
PMI at baseline [7.9 vs. 5.0]	0.87 (0.60–1.26)	0.60 (0.36–1.00)	N/A
PMI at baseline, linear effect only	N/A	N/A	0.84 (0.73–0.97)
PMI, %-change between baseline and follow-up, linear effect only	N/A	N/A	1.00 (0.99–1.01)
Age at baseline [67 vs. 53]	1.05 (0.72–1.52)	2.15 (1.16–3.99)	N/A
Stage		N/A	N/A
I or II	1 (Ref.)		
III or IV	1.52 (0.90–2.56)		
Unknown	1.48 (0.59–3.71)		
Smoking history		N/A	N/A
Never	1 (Ref.)		
Ever	1.45 (0.94–2.23)		
Sex			
Female	1 (Ref.)		
Male	1.41 (0.82–2.44)	0.94 (0.48–1.96)	
Cancer Type		N/A	N/A
Breast	0.54 (0.28–1.04)		
Lymphoma	1 (Ref.)		
Sarcoma	1.54 (0.83–2.88)		
Pre-treatment LVEF	N/A		N/A
> 55%		1 (Ref.)	
< 55%		3.12 (1.37–7.11)	

^a^Regression model: Cox regression

^b^Regression model: Competing risks regression (48 MACE)

^c^Restricted to cardiac events after follow-up CT scan (36 MACE)

## Discussion

We observed no significant association between pre-treatment (baseline) PMI and all-cause mortality. This observation is inconsistent with multiple studies that identified pre-treatment sarcopenia as an independent risk factor for overall survival in cancer patients as well as a risk factor for chemotoxicity [15, 16]. The null-association we observed in our study may be related to sample size and heterogeneity of this cohort.

Our data detected a tendency towards improved cardiac outcomes in patients with larger PMIs compared to those with smaller PMIs, which was confirmed in our exploratory (unadjusted) competing risk analysis restricted to MACE after follow-up CT. This exploratory analysis also suggested that low PMI at baseline was associated with risk of MACE. However, these analyses were limited by the small number of MACE in our cohort and should be replicated and assessed in greater detail in larger future studies.

We noticed that more frequent MACE in lymphoma may be explained by sample size difference among three types of cancer groups. There were only 29 patients with sarcoma (vs. 184 and 112 in lymphoma and breast cancer, respectively) in the MACE analysis reducing power to accurately estimate risk MACE in this patient group. Additionally, lymphoma is associated with immunocompromising conditions whose treatment may increase the risk of MACE from infection and electrolyte abnormalities.”

Sarcopenia as a predictor of cardiac events is not well studied in the cancer population treated with anthracyclines which are well-known to cause cardiotoxicity [13]. On the other hand, PMI was a powerful predictor of mortality in advanced heart failure patients undergoing left ventricular assist device (LVAD) implantation, where each unit increase in pectoralis muscle index was associated with a 27% reduction in the hazard of death after LVAD (adjusted hazard ratio, 0.73; 95% confidence interval, 0.58–0.92; *P* = 0.007) [12].

We observed that while greater PMI at baseline was associated with greater PMI at follow-up, PMI tended to decline after start of chemotherapy, and greater PMI at baseline was associated with greater relative PMI losses. The vast majority of our study population received a follow-up CT scan within a year after receiving their baseline CT scan, and the average reduction in median PMI in our study ranged between 5.6 and 17.9%, which is more than would be expected from normal aging which has been estimated at 1–2% per year in the general population past 50 years of age [17, 18].

Furthermore, after adjusting for baseline PMI, relative PMI losses tended to be higher in women than in men. This may be explained to some degree by the protective effect of testosterone in males [19]. However, investigation of underlying reasons of greater risk for PMI decline in women warrants further research.

This growing body of evidence emphasizes the importance to take into account patients’ body composition when evaluating patients newly diagnosed with cancer. Pectoralis muscle index as a strong surrogate for sarcopenia can be used as an indicator to initiate proactive measurements that may improve outcomes such as optimization of physical activity and nutritional condition before and along with anthracycline-based chemotherapy.

## Limitations

In addition to the limited number of events despite high overall mortality in this population, other limitations in our study included the setting in a single institution which limits the generalizability of our findings, and the retrospective nature of our analysis. We included two CT scans (pre- and post-chemo), but additional CT data would offer more robust findings. For our survival analysis, the date of cancer diagnosis was not available for > 20% of our study population, and thus survival time was calculated between start of chemotherapy and event of interest (death or cardiac event) or date of censoring. Furthermore, receiving two CT scans may be an indicator of greater disease severity and may not be representative of these disease populations. On the other hand, we excluded stage IV Sarcoma and breast cancer patients in our survival analyses which could have skewed our population towards lower disease severity. We detected no significant association between type of chemotherapy and PMI at follow-up; however, 88% of participants received anthracyclines (alone or in combination), which may have reduced our power to detect an association. The observed greater relative losses in PMI in those with larger PMI at baseline may in part be attributable to regression to the mean. Whereas we adjusted for receipt of anthracyclines vs. trastuzuman vs. both, we had no information on specific dosage which likely impacts the risk of adverse events, especially MACE.

While controlling for treatment in the linear regression, we had to limit the number of covariates in the survival analysis (93 deaths) and competing risk model (48 MACE). We further had no information on hypertension, diabetes, alcohol use and nutritional status such as albumin, and were hence unable to include these potential factors in our models.

## Conclusion

These data suggest that larger pre-treatment pectoralis muscle index in patients of breast cancer, lymphoma or sarcoma treated with anthracyclines tended to have lower risk of MACE. Lower baseline PMI, Female, older age and history of smoking were associated with lower pectoralis muscle after treatment. Early identification of sarcopenia using PMI could trigger proactive engagement for intervention and risk stratified therapies.

## Supplementary Information

**Additional file 1.**

## Data Availability

The datasets generated during and/or analyzed during the current study are available from the corresponding author on reasonable request.

## Abbreviations

MACE: major cardiac events
PMI: pectoralis muscle index

## References

[1] Rosenberg IH. Sarcopenia: Origins and clinical relevance. J Nutr. 1997;127(suppl 5):990S–991S. 10.1093/jn/127.5.990S 9164280

[2] Pamoukdjian F, Bouillet T, Lévy V, Soussan M, Zelek L, Paillaud E. Prevalence and predictive value of pre-therapeutic sarcopenia in cancer patients: A systematic review. Clin Nutr. 2018;37(4):1101–13. 10.1016/j.clnu.2017.07.010 28734552

[3] Srdic D, Plestina S, Sverko-Peternac A, Nikolac N, Simundic A-M, Samarzija M (2016). Cancer cachexia, sarcopenia and biochemical markers in patients with advanced non-small cell lung cancer—chemotherapy toxicity and prognostic value. Support Care Cancer..

[4] Shachar SS, Deal AM, Weinberg M (2017). Body Composition as a Predictor of Toxicity in Patients Receiving Anthracycline and Taxane-Based Chemotherapy for Early-Stage Breast Cancer. Clin Cancer Res..

[5] Cruz-Jentoft AJ, Bahat G, Bauer J (2019). Sarcopenia: revised European consensus on definition and diagnosis. Age Ageing.

[6] Mourtzakis M, Prado CMM, Lieffers JR (2008). A practical and precise approach to quantification of body composition in cancer patients using computed tomography images acquired during routine care. Appl Physiol Nutr Metab.

[7] Shen W, Punyanitya M, Wang Z (1985). Total body skeletal muscle and adipose tissue volumes: estimation from a single abdominal cross-sectional image. J Appl Physiol.

[8] Heymsfield SB, Wang Z, Baumgartner RN (1997). Human body composition: advances in models and methods. Annu Rev Nutr.

[9] Mourtzakis M, Prado CMM, Lieffers JR (2008). A practical and precise approach to quantification of body composition in cancer patients using computed tomography images acquired during routine care. Appl Physiol Nutr Metab.

[10] Kinsey CM, San José Estépar R, van der Velden J, Cole BF, Christiani DC, Washko GR. Lower pectoralis muscle area is associated with a worse overall survival in non-small cell lung cancer. Cancer Epidemiol Biomarkers Prev. 2017;26(1):38–43. 10.1158/1055-9965.EPI-15-1067 PMC5116279 27197281

[11] Jo S, Sebro R (2021). CT attenuation and cross-sectional-area index of the pectoralis are associated with prognosis in sarcoma patients. Anticancer Res.

[12] Teigen LM, John R, Kuchnia AJ, Nagel EM, Earthman CP, Kealhofer J, et al. Preoperative pectoralis muscle quantity and attenuation by computed tomography are novel and powerful predictors of mortality after left ventricular assist device implantation. Circ Heart Fail. 2017;10(9):e004069. 10.1161/CIRCHEARTFAILURE.117.004069 28912261

[13] Henriksen PA (2018). Anthracycline cardiotoxicity: an update on mechanisms, monitoring and prevention. Heart..

[14] Mullie L, Afilalo J (2019). CoreSlicer: a web toolkit for analytic morphomics. BMC Med Imaging.

[15] Aversa Z, Costelli P, Muscaritoli M (2017). Cancer-induced muscle wasting: latest findings in prevention and treatment. Ther Adv. Med Oncol..

[16] Prado CMM, Baracos VE, McCargar LJ, Reiman T, Mourtzakis M, Tonkin K (2009). Sarcopenia as a determinant of chemotherapy toxicity and time to tumor progression in metastatic breast Cancer patients receiving Capecitabine treatment. Clin Cancer Res..

[17] Sehl ME, Yates FE. Kinetics of human aging: I. rates of senescence between ages 30 and 70 years in healthy people. J Gerontol A Biol Sci Med Sci. 2001;56(5):B198–208. 10.1093/gerona/56.5.b198 11320100

[18] Hughes VA, Frontera WR, Roubenoff R, Evans WJ, Singh MAF. Longitudinal changes in body composition in older men and women: role of body weight change and physical activity. Am J Clin Nutr. 2002;76(2):473–81. 10.1093/ajcn/76.2.473 12145025

[19] Shin MJ, Jeon YK, Kim IJ (2018). Testosterone and sarcopenia. World J Mens Health.

