# Supplementary material for: Impact of pectoralis muscle loss on cardiac outcome and survival in Cancer patients who received anthracycline based chemotherapy: retrospective study
Source: BMC Cancer. 2022 Jul 13;22:763. doi: 10.1186/s12885-022-09882-w (PMC9281070; doi:10.1186/s12885-022-09882-w)
Supplement: Supplementary file 1 — Additional file 1. [file 12885_2022_9882_MOESM1_ESM.docx]

**Supplemental Table 1.** Pectoralis muscle area indexed (PMI) to height squared

|  | **Female (N=313)** | **Male (N=161)** | **P** |
| --- | --- | --- | --- |
| PMI at baseline |  |  | **<0.001** |
| Mean (SD) | 5.6 (1.7) | 8.4 (2.8) |  |
| Median (Q1,Q3) | 5.3 (4.6, 6.4) | 7.9 (6.3, 9.9) |  |
| PMI at follow-up |  |  | **<0.001** |
| Mean (SD) | 4.9 (1.3) | 7.2 (2.5) |  |
| Median (Q1,Q3) | 4.9 (4.2, 5.6) | 6.7 (5.4, 8.7) |  |
